# Can maternal postpartum testosterone and estradiol retrospectively predict the offspring's sex at birth? A cross‐sectional study in Ghana

**DOI:** 10.14814/phy2.15757

**Published:** 2023-06-23

**Authors:** Moses Banyeh, Shafiat Omotoyosi Shittu, Shamsu‐Deen Ziblim, Peter Paul Mwinsanga Dapare, Martha Nyewie, Clement Binwatin Dagungong

**Affiliations:** ^1^ Department of Biomedical Laboratory Science University for Development Studies Tamale Ghana; ^2^ Department of Population and Reproductive Health, School of Public Health University for Development Studies Tamale Ghana; ^3^ School of Public Health, Community Nurses Training School, Ministry of Health Tamale Ghana

**Keywords:** estradiol, Ghana, maternal hormones, sex at birth, testosterone

## Abstract

The selection of X‐ or Y‐bearing spermatozoa during fertilization may depend on maternal circulating sex hormones. The zona pellucida of the developing oocyte is adapted to be selective for the Y‐bearing spermatozoa when maternal circulating androgens are relatively high. This study sought to determine whether maternal postpartum testosterone and estradiol can retrospectively predict the offspring sex at birth. The study was cross‐sectional from December 2020 to April 2021 at the Reproductive and Child Health unit in Tamale. The participants were part of a previous study and comprised 178 mother–offspring dyads (mother–daughter = 90, mother–son = 88). The mothers were between the ages of 18 and 35 years and had a median (interquartile range‐IQR) postpartum interval of 111 (60–187) days. A single venous blood sample was drawn from the mothers between 8.00 am and 12.00 pm local time on each day to reduce diurnal variation. Postpartum serum estradiol, testosterone, and sex hormone‐binding globulin were assayed using the ELISA technique. The serum total testosterone and the testosterone‐to‐estradiol ratio (TT: E_2_) were higher in mothers with sons while estradiol was higher in mothers with daughters (*p* < 0.050). The total testosterone and TT: E_2_ did not markedly differ by their area under the curve (AUC: 0.91 and 0.99, respectively) but both were higher than the AUC of estradiol (0.72). The Sensitivity was 97.7%, 97.7%, and 94.5% and specificity, 88.9%, 40.0%, and 95.5% at cutoff points of >1.659 nmol/L, ≤141.862 pmol/L, and > 31.5, respectively for total testosterone, estradiol, and TT: E_2_. The maternal testosterone‐to‐estradiol ratio may be more predictive of offspring sex at birth than either testosterone or estradiol alone.

## Graphical abstract

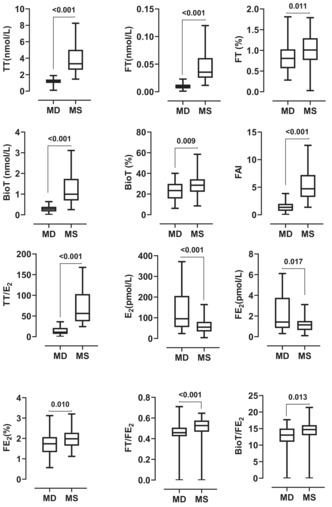

## 1 INTRODUCTION

It has been observed that the X‐ and Y‐bearing spermatozoa are produced in approximately equal numbers among mammals (James, 1998). According to the Mendelian theory of random assortment, the X‐ and Y‐bearing spermatozoa have an equal chance of fertilizing the ovum or oocyte during fertilization. If this hypothesis is true, then the sex ratio or the proportion of males at birth would be 0.5 in any given population given that the embryos of both sexes have an equal chance of survival (Barrett et al., 2020). However, the sex ratio at birth is far from 0.5 as it varies from population to population and from time to time (Cofsky, 2012). This has led some authors to suggest that since the primary sex ratio is constant, the explanation for the sex ratio deviating from 0.5 may be that there is a maternal facultative adjustment, postconception, of the primary sex ratio, through selective fetal loss, to produce the skewed secondary sex ratio (James, 2004).

The attempt to proffer an explanation for the skewness in the secondary sex ratio resulted in the widely cited Trivers and Willard hypothesis (TWH) (Trivers & Willard, 1973). The TWH posits that mammalian females produce sons when in good condition as a guarantee for offspring in the following generation, especially in polygamous settings where a male offspring in good condition have a higher chance of mating with many females to produce many offspring (Cameron, 2004). However, previous studies have failed to provide evidence of the TWH in many mammalian species (Cameron, 2004). In light of the differences in study outcomes regarding the TWH, alternative hypotheses have attempted to relate the “Condition” in the TWH to various factors including maternal dominance rank, maternal stress, maternal plasma glucose, maternal asynchrony, and maternal hormones (Cameron, 2004; Catalano & Bruckner, 2006; Grant, 1996; James, 1998; Krackow, 1995). However, these hypotheses sought to explain the secondary but not the primary sex ratio and most have been critiqued as being correlational (James, 2015).

The widely held view that the mammalian female reproductive tract was just a passive vessel for fertilization has been challenged in recent studies (Alminana‐Brines, 2015; Thurston et al., 2017). The mammalian female is capable of storing, transporting, and sorting spermatozoa using both physical, biochemical, and genetic mechanisms through the special adaptations of the reproductive tract (Thurston et al., 2017). The presence of spermatozoa in the oviduct causes the upregulation, of the oviductal epithelia, the expression of proteins capable of affecting sperm motility, storage, and viability. These phenomena regarding the active role played by mammalian females in sex selection are termed the ‘cryptic female choice (Thurston et al., 2017). The anatomical design of the female reproductive system limits, up to about 90%, the number of spermatozoa that will have access to the oocyte by allowing only spermatozoa of good morphology and viability to traverse the utero‐tubular junction (UTJ) into the oviductal isthmus (Alminana‐Brines, 2015; Holt & Fazeli, 2016; Thurston et al., 2017). This process is further aided by the activities of the cervical mucus, the vaginal pH, and polymorphonuclear cells in the UTJ leading to the oviduct. The integrity of the cell membrane and the extent of chromatin fragmentation may also determine which spermatozoa adhere to oviductal epithelia and subsequent fertilization of the oocyte (Holt & Fazeli, 2016). However, the levels of testosterone in the intratubular environment have been shown to play a major role in the selection of spermatozoa (Grant et al., 2008). Previous experimental studies in bovine and mice have demonstrated that high oviductal testosterone during the preovulatory period may adapt the zona pellucida of the developing oocyte to be selective for the Y‐bearing spermatozoa during fertilization and thus biasing the sex ratio (Epifano et al., 1995; Grant et al., 2008; Zuccotti et al., 2005). This observation may lend some credence to the follicular testosterone and the hormonal hypothesis. The ability of a mammalian female to alter her strategy of sex determination depending on the levels of androgens at every menstrual cycle or estrus has been suggested by the follicular testosterone hypothesis (Grant et al., 2008). On the contrary, the hormonal hypothesis posits that high parental testosterone (paternal and maternal) and high maternal estrogen at the time of conception favor the male zygote formation while high parental gonadotrophins and high maternal progesterone at the time of conception favor the female zygote formation (James, 2015). This is similar to the suggestion that the testosterone‐to‐estrogen ratio is a better indicator of oocyte maturity than follicle size in humans (Grant et al., 2008). Although follicular testosterone may exceed maternal blood testosterone by a factor ranging between 10,000 and 300,000, both may be correlated, since maternal dominance rank may be associated with high blood testosterone and more sons at birth (Grant et al., 2008).

An inference from the hormonal hypothesis (James, 2015) may be suggestive that the maternal testosterone‐to‐estrogen ratio would be a better predictor of sex at birth than either testosterone or estrogen alone. While a previous study in Ghana reported significant differences in maternal postpartum testosterone and estradiol by offspring sex at birth (Banyeh & Amidu, 2022), it did not compare their predictive abilities regarding sex at birth. This study, therefore, sought to determine whether maternal postpartum testosterone, estradiol, or their ratio can retrospectively predict the offspring sex at birth.

## 2 MATERIALS AND METHODS

### 2.1 Study design and population

The study was cross‐sectional between December 2020 and April 2021 and was carried out at the Reproductive and Child Health (RCH) unit in Tamale. The study population was a subsample of a previous study from which some works have been published (Banyeh et al., 2021a, b; Banyeh & Amidu, 2022). The study population comprised 178 mother–offspring dyads (mother–daughter pair = 90, mother–son pair = 88). The mothers were between the ages of 18 and 35 years and had a median (IQR) postdelivery period of 111 (60–187) days. The mothers had no known history of hormonal abnormalities and were not using hormonal contraceptives at the time of sampling.

### 2.2 Variables

The dependent or outcome variable was the sex at birth. The independent or predictor variables were maternal total testosterone and estradiol, including their indices: free and bioavailable testosterone, the free androgen index, and free estradiol. The ratios of the hormonal variables were also used as predictor variables. The variables that had confounding potential were the maternal age at the time of sampling, body mass index, and the postdelivery interval. However, the mothers with sons and those with daughters were matched by age, BMI, and postdelivery interval to reduce bias.

### 2.3 Measurements

Sociodemographic, clinical, and obstetric data were collected using an interviewer‐administered questionnaire. The standing height (to the nearest cm) was measured using a stadiometer while body weight (to the nearest 0.1 Kg) was measured using the body scale, following recommended standards. The body mass index (BMI) was calculated in Kg/m^2^. A single venous blood sample was collected into a gel separator tube between 8.00 am and 12.00 pm local time on each day to reduce diurnal variability in measurements (Beckett & Roden, 2009; Van et al., 2015). The venous blood samples were stored at 4°C to clot before being centrifuged at 3000*g* for 5 min to obtain serum. The serum samples were then aliquoted into two vials and frozen at −25°C without thawing and refreezing. The serum total testosterone estradiol and sex hormone‐binding globulin (SHBG) were measured in duplicates using the ELISA technique (Monobind Inc., Lake Forest, CA 92630, USA). The serum TT was measured using a competitive enzyme immunoassay involving a testosterone‐analog horseradish peroxidase conjugate and a biotinylated antitestosterone rabbit IgG in a streptavidin‐coated plate (product code: 3725–300). The within and between assay coefficient of variation (CV) for TT were ≤5.6% and ≤7.9%, respectively, with a sensitivity of 0.0576 ng/mL (Accuracy range: 0.29–21.9 ng/mL). Serum E_2_ was measured using a delayed competitive immunoassay involving an estradiol‐analog horseradish peroxidase conjugate and a biotinylated antiestradiol rabbit IgG in a streptavidin‐coated plate (product code: 4925–300). The sensitivity of the test kit was 8.2 pg/mL with an accuracy range of 10–4300 pg/mL. The within and between assay coefficient of variation (CV) of the E2 test kit were ≤7.5% and ≤8.2%, respectively. The SHBG in serum samples were measured using an immunoenzymometric assay involving an antihuman SHBG‐horseradish peroxidase conjugate and a biotinylated antihuman IgG in a streptavidin‐coated plate (product code: 9125–300). The test kit had a sensitivity of 0.0122 nmol/L within a 4.6–184.0 nmol/L accuracy range. The intra‐assay precision of the test kit ranged from 1.5% to 2.6%. Calibration curves were created using manufacturer‐supplied calibrators and then validated using manufacturer‐supplied control samples. The serum albumin was measured on the BT 1500 automated biochemistry analyzer (Biotechnica Instruments, SPA, Italy) using the manufacturer‐recommended reagents, calibrators, and controls. The free estradiol was calculated following a recommended formula by Södergard, Bäckström (Södergard et al., 1982) from the website (https://hrt.cafe/free‐e2‐estimator/). Similarly, the serum albumin was used in the calculation of the free and bioavailable testosterone based on a recommended formula by Vermeulen, Verdonck (Vermeulen et al., 1999) from the website (http://www.issam.ch/freetesto.html). The free androgen index (FAI) was calculated as the ratio of the total testosterone and SHBG (all in nmol/L). The ratio variables were then calculated from the estimated variables in the same unit or converted to the same unit before the calculation. The samples were not blinded before analyses.

### 2.4 Statistical analysis

The data were analyzed using SPSS (v23) and MedCalc (v14.8) statistical software. The normality of the data was determined using the Shapiro–Wilk test. The data were stratified by offspring sex and were then summarized as median (IQR). The differences between mother–daughter and mother–son dyads were determined using the Mann–Whitney U test. The data were stratified by days after delivery and then presented as box and whisker plots. The predictive abilities of the hormonal variables in the determination of the offspring sex at birth were performed using the receiver operator characteristic curve (ROC) based on the Hanley and McNeil method (McNeil & Hanley, 1984). The best cutoff point was determined at the Youden index and the corresponding sensitivity, specificity, likelihood ratio, and the area under the curve (AUC) were documented as well as their 95% confidence intervals. All the statistical analyses were two‐sided at *p* value <0.050 considered statistically significant.

### 2.5 Ethics statement

The study complied with the guidelines for human subject studies as proposed by the Declaration of Helsinki (1964) and its later amendments. Ethical approval for this study was obtained from University for Development Studies Institutional Review Board (UDS/RB/003/21). Written informed consent was obtained from all subjects before the study. The study was voluntary and a participant could opt out at any stage of the study. Trial registration‐Not applicable.

## 3 RESULTS

### 3.1 General characteristics

The characteristics of the study population are summarized in Table 1. The study population had a postpartum interval between 7 and 397 days. The majority of the mothers were married (94.4%) and identified with the Islamic religion (85.0%) but the majority were also unemployed (55.0%).

**TABLE 1:** The general characteristics of the study population.

Variable	Min‐max/*n* (%)
Age (years)	18–36
Postpartum interval (days)	7–397
Height (cm)	149–176
Weight (Kg)	45.3–83.1
BMI (Kg/m^2^)	17.3–33.4
Cultural group	
Mole‐Dagomba	170 (96.0)
Others	8 (4.0)
Religious group	
Islam	152 (85.0)
Others	26 (15.0)
Educational status	
None	22 (12.4)
Basic	38 (21.3)
Secondary	70 (39.3)
Tertiary	48 (27.0)
Employment status	
None	98 (55.0)
Self	54 (30.3)
Salary	26 (14.7)
Marital status	
Married	168 (94.4)
Others	30 (5.6)
Offspring sex at birth	
Daughters	90 (50.6)
Sons	88 (49.4)

*Note*: The continuous variables were summarized as minimum‐maximum while the categorical variables were summarized as number (percent).

### 3.2 Comparison of maternal hormonal variables

**FIGURE 1:**
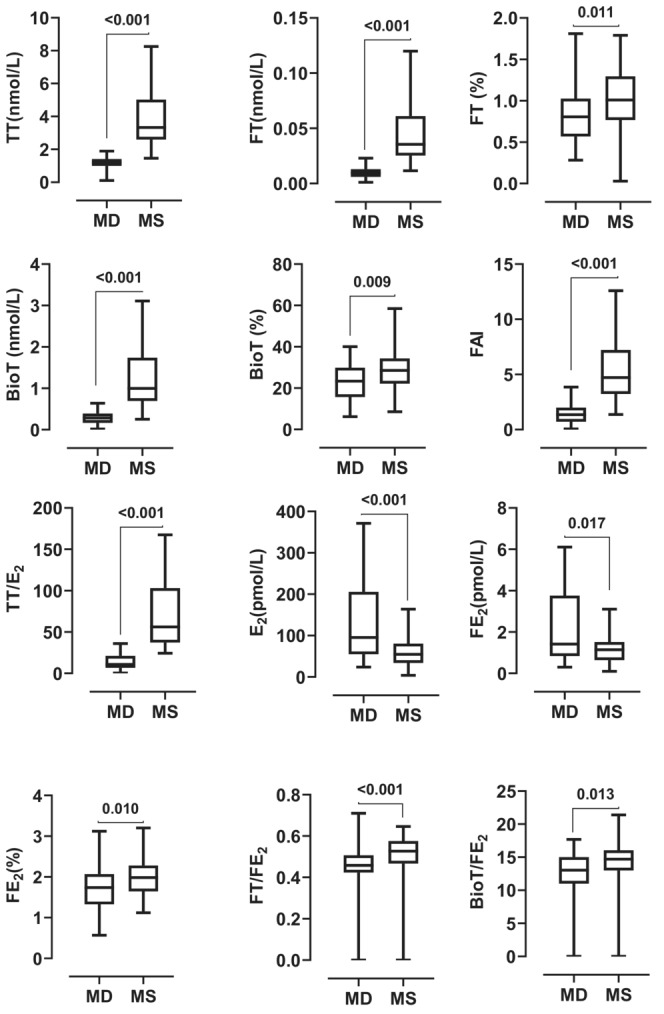
Box and whisker plots comparing circulating hormones of mothers with daughters (MD) and mothers with sons (MS). BIOT, bioavailable testosterone; E_2_, estradiol; FAI, free androgen index; FE_2_, free estradiol; FT, free testosterone; TT, total testosterone.

**FIGURE 2:**
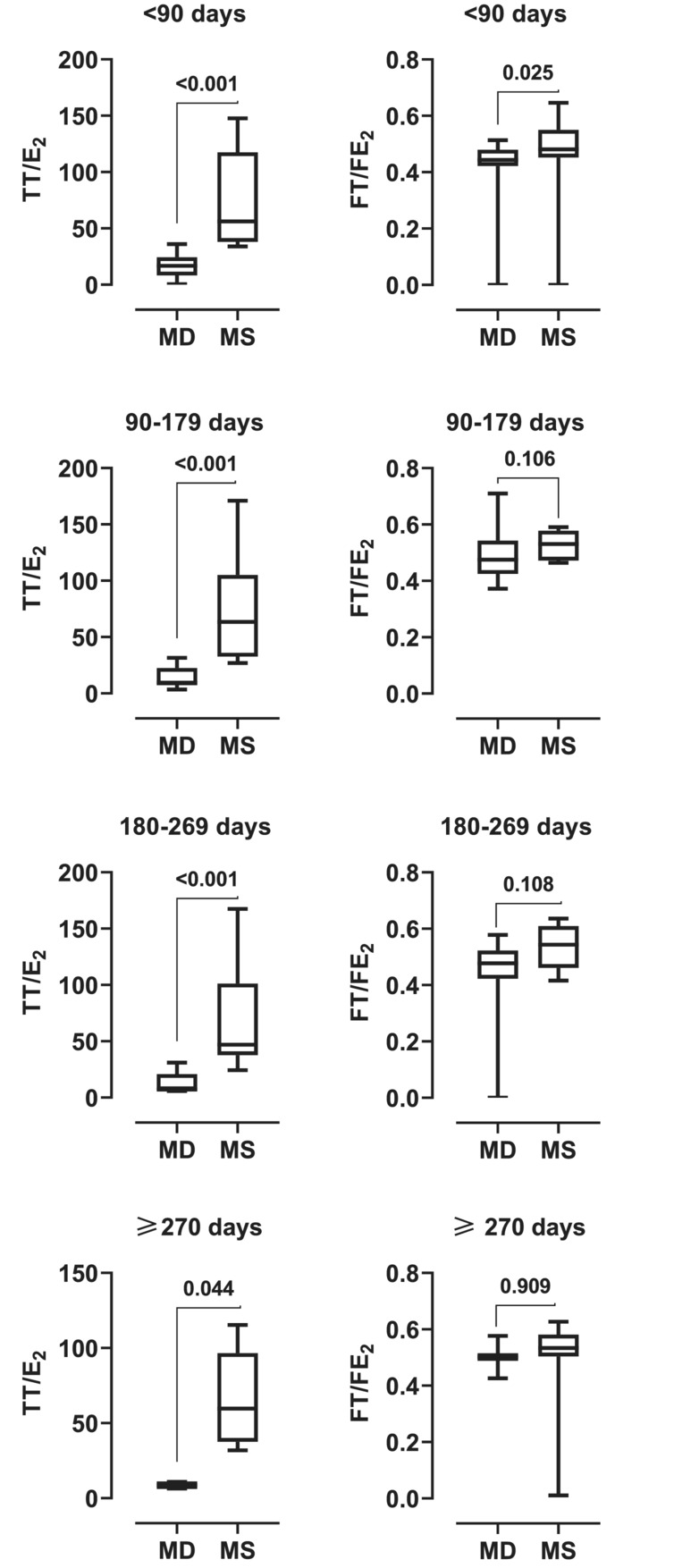
Box and whisker plots comparing the testosterone‐to‐estradiol ratio of mothers with daughters (MD) and mothers with sons (MS) stratified by days after delivery. E_2_, estradiol; FE_2_, free estradiol; FT, free testosterone; TT, total testosterone.

The hormonal variables of the mothers with sons and those with daughters were compared in Table 2, Figures 1 and 2. The mothers with daughters and those with sons did not differ in their BMI and age at the time of sampling. However, mothers with sons had significantly higher levels of total, free, and bioavailable testosterone than those with daughters (*p* < 0.050). Although mothers with daughters had significantly higher levels of total and free estradiol than those with sons, the opposite was true for percent free estradiol and the testosterone‐estradiol ratio (*p* < 0.050). When the sample was stratified into days after delivery, the testosterone‐to‐estradiol ratio was consistently higher in mothers with sons than in mothers with daughters in all strata.

**TABLE 2:** Comparison of variables of the mothers with daughters and the mothers with sons.

Variable	Mothers (daughters)	Mothers (sons)	*p* value
Age (years)	23.0 (21.0–25.0)	25.0 (20.0–27.0)	0.065
BMI (Kg/m2)	22.8 (20.5–25.6)	22.9 (20.8–26.0)	0.493
Postdelivery (days)	111 (67–157)	101 (53–210)	0.392
ALB (g/L)	51.4 (45.8–58.0)	51.3 (47.7–55.0)	0.871
SHBG (nmol/L)	92.0 (67.9–149.0)	71.2 (51.2–106.1)	0.011
TT (nmol/L)	1.20 (0.98–1.41)	3.45 (2.65–5.06	<0.001
FT (nmol/L)	0.009 (0.006–0.013)	0.036 (0.025–0.062	<0.001
FT (%)	0.81 (0.56–1.01)	1.01 (0.77–1.27)	0.011
BIOT (nmol/L)	0.27 (0.15–0.39)	1.00 (0.68–1.88)	<0.001
BIOT (%)	23.4 (15.7–29.5)	28.6 (21.8–34.6)	0.009
FAI	1.35 (0.69–1.89)	4.72 (3.22–7.33)	<0.001
TT: E_2_	10.25 (6.95–21.70)	56.2 (37.90–104.5)	<0.001
E_2_ (pmol/L)	90.9 (54.0–207.9)	54.7 (34.2–82.0)	<0.001
FE_2_ (pmol/L)	1.42 (0.83–3.80)	1.15 (0.64–1.52)	0.017
FE2 (%)	1.72 (1.33–2.04)	1.99 (1.63–2.26)	0.010
FT:FE_2_	0.46 (0.42–0.51)	0.53 (0.47–0.58)	0.001
BIOT:FE_2_	13.2 (11.0–15.0)	14.7 (12.9–16.2)	0.013

Abbreviations: ALB, albumin; BIOT, bioavailable testosterone; BMI, body mass index; E_2_, estradiol; FAI, free androgen index; FT, free testosterone; TT, total testosterone; FE_2_, free estradiol.

*Note*: The results were summarized as median (IQR). The variables of the mothers with daughters and the mothers with sons were compared using the Mann–Whitney U Test.

### 3.3 Predictive abilities of maternal postpartum hormonal variables

**FIGURE 3:**
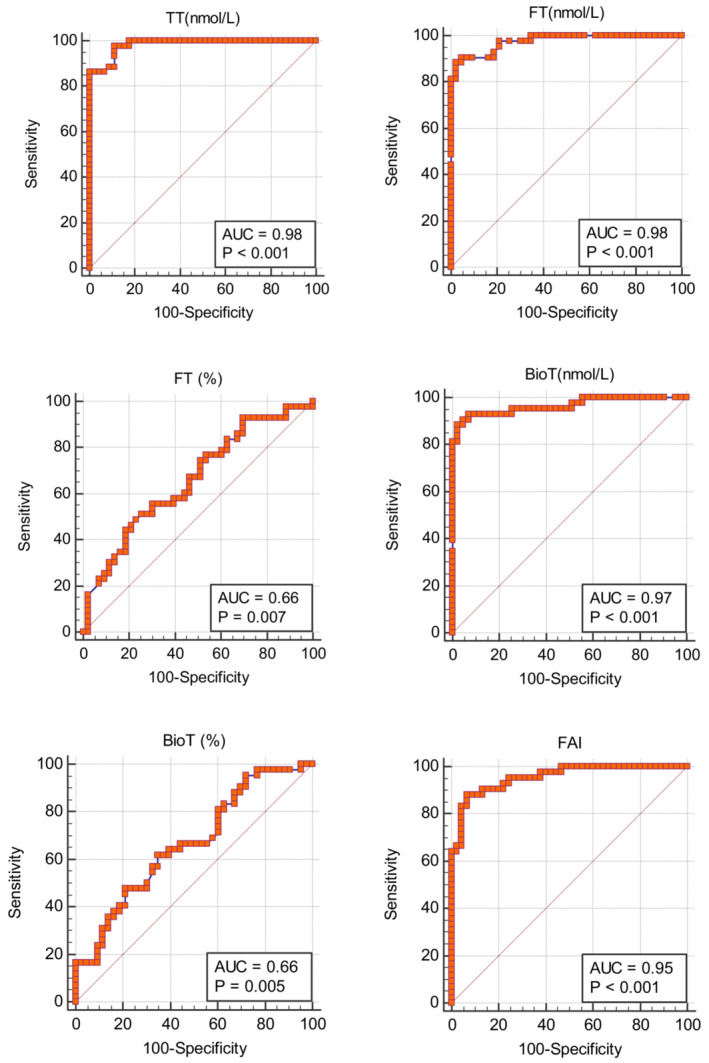
Receiver operator characteristics plots show the area under the curve (AUC) in predicting offspring sex at birth using maternal postpartum hormones. BIOT, bioavailable testosterone; FAI, free androgen index; FT, free testosterone; TT, total testosterone.

**FIGURE 4:**
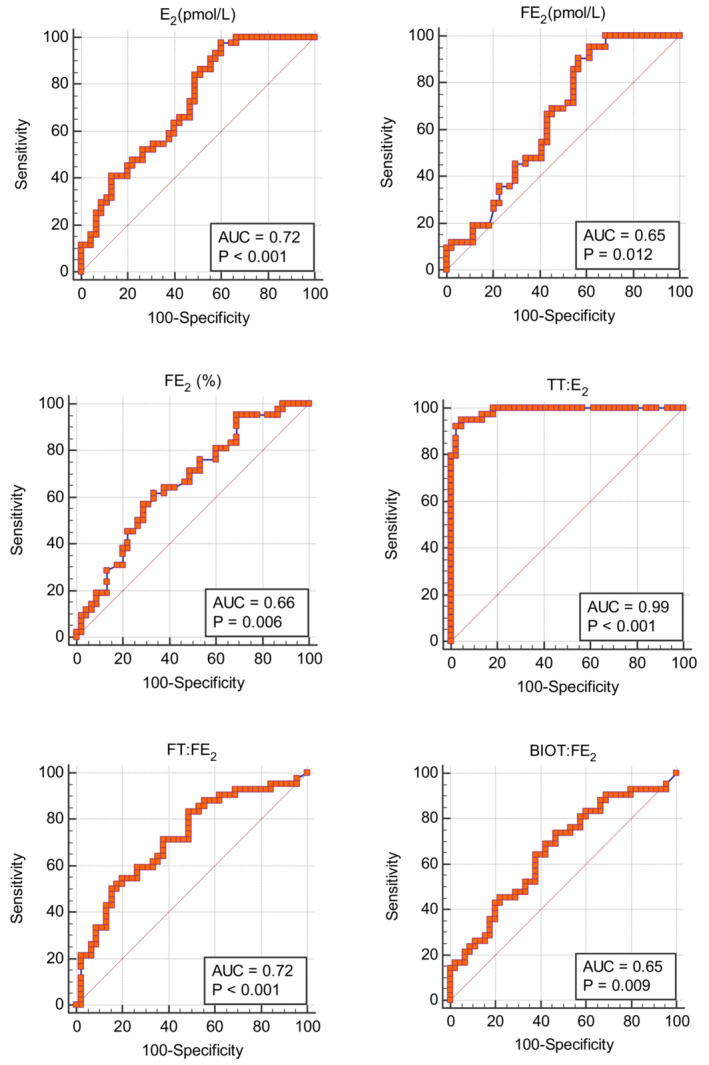
Receiver operator characteristics plots showing the area under the curve (AUC) in predicting offspring sex at birth using maternal postpartum hormones. BIOT, bioavailable testosterone; E_2_, estradiol; FE_2_, free estradiol; FT, free testosterone; TT, total testosterone.

The predictive abilities of maternal hormones of the offspring sex at birth are summarized in Table 3 and Figures 3 and 4. The total testosterone, the free and bioavailable testosterone, the free androgen index, and the testosterone‐to‐estradiol ratio had AUCs ≥0.95 without significant differences between them. However, the testosterone‐estradiol ratio had the highest AUC of 0.99 with a sensitivity and specificity of 94.9% and 95.5%, respectively. Moreover, the testosterone‐estradiol ratio had the highest positive likelihood ratio of 20.9.

**TABLE 3:** Predictive abilities of maternal postpartum hormones of the offspring sex at birth.

Variable	Cutoff point	Sensitivity (95 % CI)	Specificity (95 % CI)	+LR (95% CI)	— LR (95 % CI)
TT (nmol/L)	>1.659	97.7 (88.0–99.9)	88.9 (75.9–96.3)	8.8 (3.8–20.1)	0.0 (0.0–0.2)
FT (nmol/L)	>0.0186	90.7 (77.9–97.4)	95.4 (84.2–99.4)	19.5 (5.0–75.7)	0.1 (0.0–0.2)
FT (%)	>0.939	55.8 (39.9–70.9)	69.8 (53.9–82.8)	1.9 (1.1–3.1)	0.6 (0.4–0.9)
BIOT (nmol/L)	>0.498	93.0 (80.9–98.5)	93.0 (80.9–98.5)	13.3 (4.5–39.8)	0.1 (0.0–0.2)
BIOT (%)	>26.3	61.9 (45.6–76.4)	65.1 (49.1–79.0)	1.8 (4.5–39.8)	0.6 (0.4–0.9)
FAI	>2.734	88.1 (74.4–96.0)	93.3 (81.7–98.6)	13.2 (4.4–39.7)	0.1 (0.4–0.9)
E_2_ (pmol/L)	≤141.862	97.7 (88.0–99.9)	40.0 (25.7–55.7)	1.6 (1.3–2.1)	0.1 (0.0–0.4)
FE_2_ (pmol/L)	≤2.69	95.2 (83.8–99.4)	38.6 (24.4–54.5)	1.6 (1.2–2.0)	0.1 (0.0–0.5)
FE_2_ (%)	>1.85	61.9 (45.6–76.4)	66.7 (51.0–80.0)	1.9 (1.2–3.0)	0.6 (0.4–0.9)
TT: E_2_	>31.5	94.9 (82.7–99.4)	95.5 (84.5–99.4)	20.9 (5.4–81.0)	0.1 (0.0–0.2)
FT: FE_2_	>0.5076	54.8 (38.7–70.2)	80.0 (65.4–90.4)	2.7 (1.4–5.2)	0.6 (0.4–0.8)
BIOT: FE_2_	>13.268	73.8 (58.0–86.1)	53.3 (37.9–68.3)	1.6 (1.1–2.3)	0.5 (0.3–0.9)

Abbreviations: ALB, albumin; BIOT, bioavailable testosterone; BMI, body mass index; CI, confidence interval; E_2_, estradiol; FAI, free androgen index; FE_2_, free estradiol; FT, free testosterone; LR, likelihood ratio; TT, total testosterone.

*Note*: Receiver operator characteristics show the predictive ability of maternal hormones for the sex at birth.

## 4 DISCUSSION

The study aimed to determine whether maternal postpartum testosterone, estradiol, or their ratio can retrospectively predict the offspring sex at birth. It was observed that mothers with sons had significantly higher total, free, and bioavailable testosterone than those with daughters and vice versa for total estradiol. The testosterone‐to‐estradiol ratio was also significantly higher in mothers with sons than those with daughters. While testosterone was better than estradiol in predicting offspring sex at birth, the positive likelihood ratio was markedly higher for the testosterone‐to‐estradiol ratio than for the individual hormones.

Mothers with sons had significantly higher serum testosterone than mothers with daughters. This finding may be consistent with the hormonal hypothesis (James, 2015). However, it should be stated that the hormonal hypothesis refers to high testosterone at the time of conception. The current study used postpartum hormonal levels which may be correlated with preconception levels. Another indirect source of evidence in support of the hormonal hypothesis is the observation from previous studies which showed that the proportion of sons at birth is higher among women who were carriers of the hepatitis B virus or were infected with *Toxoplasma gondii* at the time of conception (Flegr & Kaňková, 2020; James, 2008; Kaňková et al., 2007). It has been established that Toxoplasmosis and HBV infection is characterized by increased testosterone levels (James, 2008). Although the studies point to increased testosterone at the time of conception, postpartum testosterone may still be high among women who are seropositive for *T. gondii* or HBV at the time of conception if left untreated. It has been hypothesized that the parental (father or mother or both) testosterone and maternal estrogen at the time of conception are supportive of male zygote development (James, 2015).

However, the findings of the current study were at variance with previous studies that did not find any significant difference in maternal testosterone between mothers with sons and those with daughters (Nabi et al., 2014; Ventura et al., 2013). There are methodological differences between the current study and the studies by Ventura, Gomes (Ventura et al., 2013) and Nabi, Aziz (Nabi et al., 2014); maternal blood samples were collected postpartum in the current study while in the previous studies, maternal blood was collected in the second trimester of pregnancy. Although perinatal and postpartum serum testosterone may still be correlated, postpartum changes are possible (Králík et al., 2019; Rousso et al., 2002). Another significant difference is that albeit both studies involved singleton pregnancies, only first‐time mothers were recruited in the current study but it was not explicitly stated whether this was the case in the previous studies. According to the physiologic regression hypothesis, the interbirth intervals (IBI) may have an impact on subsequent pregnancies after a previous pregnancy as there are significant changes in maternal sex hormones during pregnancy (Rousso et al., 2002). When IBI are longer, a mother regresses to her primigravid state but when the IBI is shorter the mother would not have recovered fully from the impact of pregnancy which may include hormonal changes (Rousso et al., 2002). Mothers with a previous history of delivery may confound the offspring's sex at birth due to the physiological changes emanating from the pregnancy and this may include the maternal hormonal environment. Further evidence of the impact of a previous pregnancy on subsequent siblings has been determined in previous studies that showed that children with older male siblings had a lower 2D:4D ratio than controls although this has not been a universal observation (Králík et al., 2019; Saino et al., 2006). The 2D:4D ratio is a negative and positive correlate of prenatal androgen and estrogen exposure, respectively (Manning & Trivers, 2002; Zheng & Cohn, 2011).

The predictive abilities of the total, free, and bioavailable testosterone were better than estradiol given their larger area under the curve (Akobeng, 2007). Although the AUC of the testosterone‐to‐estradiol ratio was not significantly different from those of testosterone or its free forms, the higher positive likelihood ratio for sex prediction for testosterone‐to‐estradiol ratio and free testosterone makes them better predictors of offspring sex than the rest. Free testosterone, unlike the bound form, is capable of acting intracellular and a free fraction of testosterone may be accounting for the predictive ability of the total testosterone. The likelihood ratio is a better determinant of a test's predictive ability than either the sensitivity, specificity, or predictive values as it is less prone to confounding factors such as population variability (Chu, 1999; Cook, 2007). Because the likelihood ratio is the ratio of sensitivity to specificity, it is not affected by population variabilities (Attia, 2003; Chu, 1999). The larger the positive LR (>10) and the smaller the negative LR (<0.1) the better a test's predictive ability (Ranganathan & Aggarwal, 2018). This observation that the testosterone‐to‐estradiol ratio may be a better determinant of the offspring sex at birth may offer some support for the hormonal hypothesis which suggested that high maternal testosterone and estradiol at the time of conception favors male zygote formation and development (James, 2015). The hormonal hypothesis may partly account for the determination of the primary sex ratio as there are further oocyte–spermatozoa interactions including immunologic and genetic (Thurston et al., 2017).

While both testosterone and estradiol may play roles in sex selection during fertilization, available literature points more to testosterone. The levels of intrafollicular testosterone during the development of the oocyte have an effect on the oocyte's zona pellucida regarding its being fertilized by either X‐ or Y‐bearing spermatozoa (Cameron et al., 2017). It has been shown that oocytes that develop in high testosterone follicular environments are more likely to be fertilized by Y‐bearing spermatozoa (Cameron et al., 2017). When controlled for estradiol, high intrafollicular testosterone still supported male zygote formation, an indication that testosterone but not estrogen is the major contributor to offspring sex at fertilization although the testosterone‐to‐estrogen may be a better indicator of follicular development (Grant et al., 2008). Although it is not clear how follicular testosterone influences the selection of the X‐ or Y‐bearing spermatozoa by the oocyte, it is suggested that testosterone may alter protein expression (e.g., tetraspanin CD9) on the zona pellucida of the oocyte or alter the interaction between spermatozoa and the cumulus cells that surround the oocyte in a way that favors the Y‐bearing spermatozoa (Gadella & Evans, 2011; Grant et al., 2008). It has also been observed that follicular testosterone exceeds maternal blood testosterone by a factor ranging between 10,000 and 300,000. However, since maternal dominance rank may be associated with sons at birth and high blood testosterone, maternal blood and follicular testosterone may be positively correlated (Grant et al., 2008). However, previous studies have failed to report any significant effect of intrafollicular testosterone on sex at birth (Cameron et al., 2017; García‐Herreros et al., 2010). This has led to the suggestion that any observable changes in the intrafollicular testosterone concentration may be a consequence rather than the cause of the sex at conception (Cameron et al., 2017).

The current study is among a few such studies to come from Ghana to have determined the comparative abilities of maternal postpartum hormones in the prediction of the offspring sex at birth. In this study, not only crude testosterone or estradiol were used but also their free forms and their ratios. The authors, however, acknowledge that sampling maternal blood in the first or second trimester will be more reflective of preconception hormonal levels than postpartum samples which may be subject to changes (Rousso et al., 2002). Further studies are recommended that will address the limitation of the current study.

## 5 CONCLUSION

Maternal postpartum hormones may still be reflective of the offspring's sex at birth. Although testosterone is a better predictor of the offspring sex than estradiol, their ratio may be more reliable than either hormone alone. This may due to the suggestion that high maternal testosterone and estrogen at the time of conception favors the male zygote formation. These findings imply a preconception sexual selection strategy.

## AUTHOR CONTRIBUTIONS

MB and SDZ were involved in conceptualization, project leadership, project management, methodology, validation, and writing—revisions. CBD, SOS and MN were involved in data collection, sample analysis, and writing—revisions. MB and PPMD were involved in data analysis, interpretation, writing—the initial draft; writing—revisions.

## FUNDING INFORMATION

This study received no funding.

## CONFLICT OF INTEREST STATEMENT

The authors wish to declare no financial or conflict of interest in conducting this study.

## Data Availability

The data supporting the findings of this study will be made available upon reasonable request from the corresponding author.
